# Improved prostate delineation in prostate HDR brachytherapy with TRUS‐CT deformable registration technology: A pilot study with MRI validation

**DOI:** 10.1002/acm2.12040

**Published:** 2017-01-19

**Authors:** Xiaofeng Yang, Peter J. Rossi, Ashesh B. Jani, Hui Mao, Zhengyang Zhou, Walter J. Curran, Tian Liu

**Affiliations:** ^1^ Department of Radiation Oncology and Winship Cancer Institute Emory University Atlanta GA USA; ^2^ Department of Radiology and Imaging Sciences and Winship Cancer Institute Emory University Atlanta GA USA; ^3^ Department of Radiology Nanjing Drum Tower Hospital Nanjing China

**Keywords:** CT, HDR brachytherapy, prostate contour, transrectal ultrasound (TRUS), TRUS‐CT registration

## Abstract

Accurate prostate delineation is essential to ensure proper target coverage and normal‐tissue sparing in prostate HDR brachytherapy. We have developed a prostate HDR brachytherapy technology that integrates intraoperative TRUS‐based prostate contour into HDR treatment planning through TRUS‐CT deformable registration (TCDR) to improve prostate contour accuracy. In a perspective study of 16 patients, we investigated the clinical feasibility as well as the performance of this TCDR‐based HDR approach. We compared the performance of the TCDR‐based approach with the conventional CT‐based HDR in terms of prostate contour accuracy using MRI as the gold standard. For all patients, the average Dice prostate volume overlap was 91.1 ± 2.3% between the TCDR‐based and the MRI‐defined prostate volumes. In a subset of eight patients, inter and intro‐observer reliability study was conducted among three experienced physicians (two radiation oncologists and one radiologist) for the TCDR‐based HDR approach. Overall, a 10 to 40% improvement in prostate volume accuracy can be achieved with the TCDR‐based approach as compared with the conventional CT‐based prostate volumes. The TCDR‐based prostate volumes match closely to the MRI‐defined prostate volumes for all 3 observers (mean volume difference: 0.5 ± 7.2%, 1.8 ± 7.2%, and 3.5 ± 5.1%); while CT‐based contours overestimated prostate volumes by 10.9 ± 28.7%, 13.7 ± 20.1%, and 44.7 ± 32.1%. This study has shown that the TCDR‐based HDR brachytherapy is clinically feasible and can significantly improve prostate contour accuracy over the conventional CT‐based prostate contour. We also demonstrated the reliability of the TCDR‐based prostate delineation. This TCDR‐based HDR approach has the potential to enable accurate dose planning and delivery, and potentially enhance prostate HDR treatment outcome.

## 1 Introduction

High‐dose‐rate (HDR) brachytherapy has been established as an effective treatment for localized prostate cancer over the past two decades.^1^, ^2^ Modern HDR prostate brachytherapy, utilizing the most advanced imaging and computer technology, is able to provide high levels of local and biochemical control for intermediate‐ to high‐risk prostate cancers.^3^, ^4^, ^5^ There is a consensus today that transrectal ultrasound (TRUS) guided CT‐based HDR brachytherapy is most common approach for prostate HDR brachytherapy.^6^, ^7^, ^8^, ^9^, ^10^ However, one of the main challenges of CT‐based HDR brachytherapy is to accurately contour the prostate in CT images due to the poor soft‐tissue contrast.^11^, ^12^

We have recently developed an approach to improve the accuracy of the prostate delineation utilizing intraoperative TRUS‐based prostate contour and TRUS‐CT deformable registration (TCDR).^13^, ^14^ Studies have shown that CT‐based prostate contours often overestimate the prostate volumes by over 30%, due to the following issues: tendency to include portions of neurovascular bundles; poor definition of the interface between the posterior prostate edge and the anterior rectal wall; and difficulties distinguishing the lower limit of the prostate apical region because of its close proximity to the pelvic floor muscles and the poor contrast between these two soft tissues.^15^ To overcome the inaccuracy of CT‐based prostate contour, we propose to incorporate TRUS‐based prostate contour, which has been shown to provide accurate prostate volumes.^16^, ^17^, ^18^, ^19^ Since HDR catheter insertions are guided by intraoperative TRUS, 3D TRUS images of the prostate can be acquired in the OR, which can be easily integrated into the prostate HDR workflow. Since CT has the advantage of accurate dose calculation and HDR catheter recognition, the TRUS‐based prostate contour is transformed onto the CT images using TRUS‐CT image registration for dose calculation in the treatment planning. Combining the strength of the CT and TRUS, the TCDR‐based HDR approach could represent a substantial improvement in terms of tumor targeting and normal‐tissue sparing in prostate HDR brachytherapy.

In this report, we will describe the workflow of the TCDR‐based HDR prostate brachytherapy. The objectives of this study are two folds: (a) to test the clinical feasibility of the TCDR‐based HDR prostate brachytherapy workflow, and (b) to investigate the performance of the TCDR‐based prostate HDR approach in terms of prostate contour accuracy and reliability as compared with the conventional CT‐based HDR procedure.

## 2 Methods

### 2.A Patient and radiotherapy characteristics

In this retrospective clinical study, imaging data from 16 patients were used to test the feasibility and performance of the TCDR‐based prostate delineation. All patients (age: 65.5 ± 7.3) had received conventional CT‐based HDR brachytherapy for localized prostate cancer between January 2013 and September 2013. Among the 16 patients, seven patients received HDR brachytherapy as monotherapy (total dose 27 Gy, 13.5 Gy/fraction) and nine patients received HDR brachytherapy as boost (total dose 19 Gy, 9.5/fraction) in combination with external beam radiation therapy (total dose 45 Gy, 1.8/fraction).

### 2.B Patient Imaging – MRI, TRUS and CT scans

All patients enrolled received MRI, TRUS, and CT scans of the prostate. All patients had diagnostic MR scans prior to the HDR procedures. In this study, we used prostate contours from the MR images as the gold standard to evaluate the TCDR‐based prostate delineation. As compared with CT images, MRI has high soft tissue contrast and clear prostate boundaries.^20^ The 3D intraoperative TRUS scan of the prostate was obtained right after the catheter insertions in the operating room and the TRUS images were used for the prostate contour. The CT scan followed the conventional CT simulation protocol for HDR treatment planning. The specific parameters of the MRI, TRUS, and CT scans are described below.

#### 2.B.1 MRI scan

All patients were scanned in feet‐down supine position with a body coil using a 1.5T Philips MRI with a voxel size of 1.00 × 1.00 × 2.00 mm^3^. All prostates were manually segmented from the T2‐weighted MR images by an experienced radiation oncologist. To evaluate the performance of the TCDR‐defined prostate contour technology, we compared the TCDR‐based prostate contours with MRI‐defined prostate contours.

#### 2.B.2 TRUS scan

The patient is scanned in the lithotomy position and a series of parallel axial (transverse) scans are captured from the apex to the base with a 2 mm step size to cover the entire prostate gland plus 5 to 10 mm anterior and posterior margins. For a typical prostate, 30 to 40 TRUS images would cover 60 to 80 mm in the longitudinal direction. In this study, all patients were scanned in the lithotomy position using a HI VISION Avius ultrasound machine (Hitachi Medical Group, Japan) and a 7.5 MHz prostate bi‐plane probe (UST‐672‐5/7.5). The transrectal ultrasound probe was held with a mechanical SurePoint stepper (Bard Medical, Inc., Covington, GA, USA) to allow for a manual stepwise movement along the longitudinal axis. The TRUS voxel size was 0.12 × 0.12 × 1.00 mm^3^ for seven patients and 0.12 × 0.12 × 2.00 mm^3^ for the remaining nine patients. A radiation oncologist subsequently contours the prostate volumes using these 3D TRUS prostate images. In general, it takes 5 to 10 minutes to contour a prostate volume. Although this may be time consuming, physician's manual contour of the prostate are still the standard practice in the clinic.

#### 2.B.3 CT scan

For the treatment planning CT images, all patients were scanned in feet‐down supine position using a Philips CT scanner (Philips Healthcare, Andover, MA, USA). The CT voxel size was 0.68 × 0.68 × 1.00 mm^3^.

### 2.C TCDR‐based prostate HDR brachytherapy workflow

**Figure 1:**
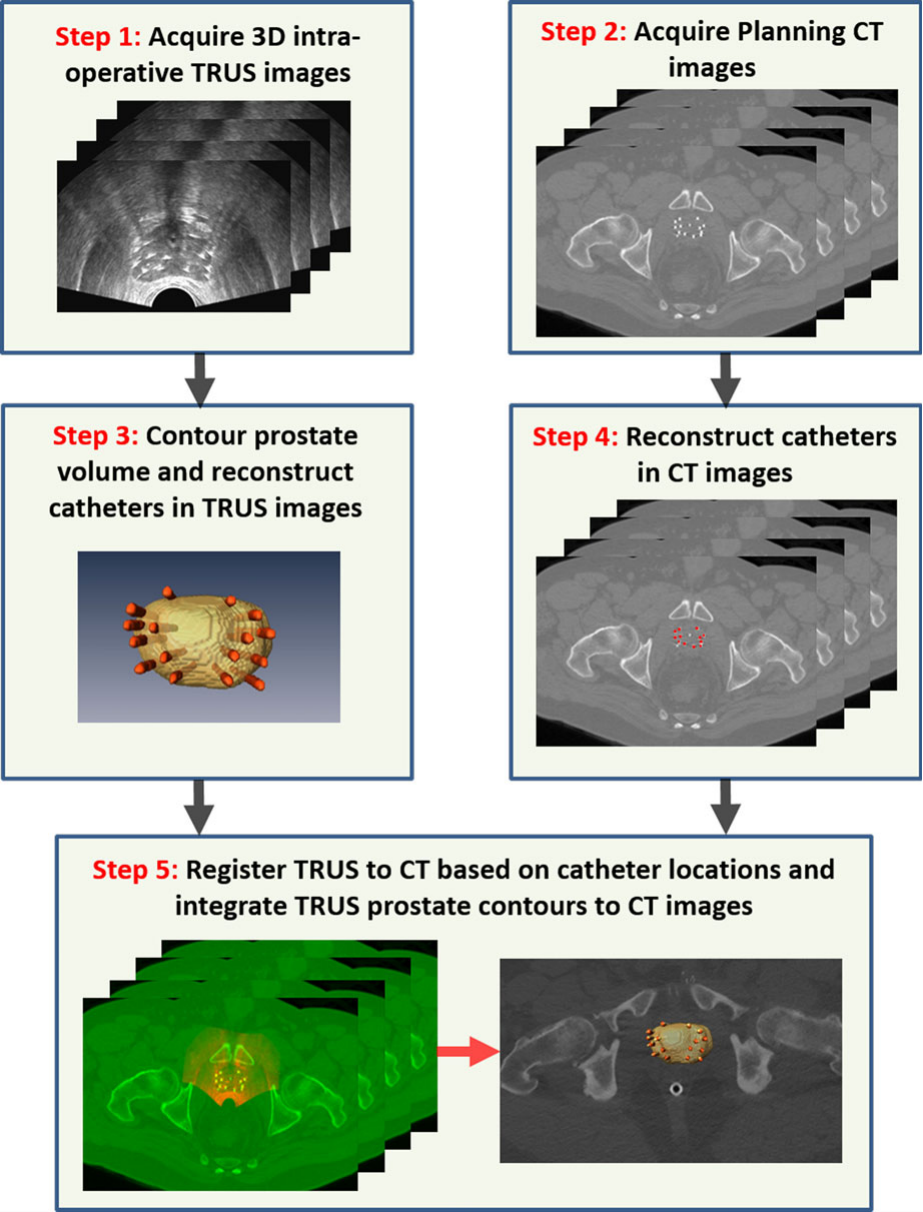
Flowchart of integrating TRUS‐based prostate volume into CT‐based HDR treatment planning.

Figure 1 shows the step‐by‐step workflow. Overall, the TCDR‐based HDR technique follows the American Brachytherapy Society consensus guidelines for HDR prostate brachytherapy.^21^ The prostate HDR process begins similarly to that for conventional CT‐based HDR in terms of bowel prep, patient positioning, and the use of TRUS. Three gold markers were placed at the base, middle or apex of the prostate under the TRUS guidance. The five major steps of the TCDR‐based prostate segmentation are the following: (a) The 3D TRUS prostate images are captured after the catheter insertions during the HDR procedure; (b) A post‐operative CT scan (CT simulation) is obtained for dose calculation; (c) The prostate volume is contoured in the TRUS images; (d) The HDR catheters in the 3D TRUS and CT images are reconstructed; (e) The TRUS‐CT image registration is performed using HDR catheters as landmarks, and the TRUS‐based prostate volume is integrated into the 3D CT images for HDR treatment planning. In this patient group, 12–16 catheters (mean ± STD: 15.1 ± 1.7) were implanted. After TRUS‐CT image registration, the planning system generates a treatment plan, indicating desired locations for treatment catheters, relative treatment times for the dwell positions, and the resulting dose distribution. Before treatment delivery, the catheter positions are checked again to make sure no catheter movements during CT simulation and treatment planning period. Once satisfactory catheter placement has been confirmed, an iridium‐192 source is used to deliver the HDR treatment.

### 2.D Inter‐ and intra‐observer reliability of TCDR‐based and CT‐based prostate contour

Three observers participated in an inter‐ and intra‐observer reliability of TCDR‐based and CT‐based prostate contours in a subset of eight patients using Oncentra Brachytherapy planning system (Elekta, Stockholm, Swedish). Observer 1 is the treating radiation oncologist with 15‐year experience. Observer 2 is a radiation oncologist with 20‐year experience. Observer 3 is a radiologist with 20‐year experience. To evaluate inter‐observer reliability of the prostate contours, three observers performed CT‐based prostate contours as well as the TRUS‐based prostate contours which were used to generated TCDR‐based prostate contours. Each observer was blinded to other observers’ contours. The variations of the CT‐based and TCDR‐based prostate volumes were calculated for the assessment of inter‐observer reliability. To evaluate intra‐observer reliability of the prostate contours, observer 1 performed CT‐based and the TRUS‐based prostate contours twice. The time between the first and second contours was over 3 months, which was long enough to reduce recall bias. Again, the variations of the CT‐based and TCDR‐based prostate volumes were calculated for the assessment of intra‐observer reliability.

## 3 Results

### 3.A Accuracy of TCDR‐based prostate contours

**Figure 2:**
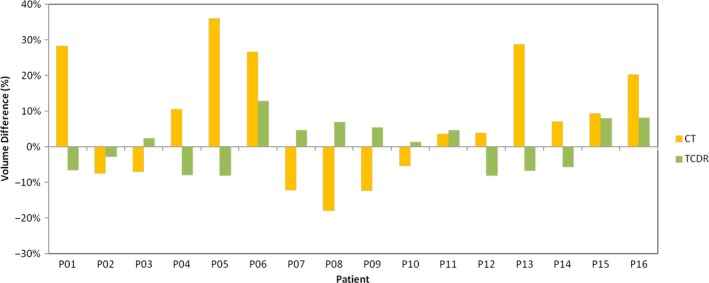
Prostate volume difference of the TCDR‐based and CT‐based contours, as compared with the MR‐defined prostate contours.

Figure 2 shows the prostate volume differences in the TCDR‐based and CT‐based prostate contours as compared with the MR‐defined prostate contours for all 16 patients. The TRUS, CT, and MRI prostate contours were delineated by the radiation oncologist (observer 1) who had treated all 16 patients. There is no significant difference (*P* = 0.54) between the TCDR‐based and MRI‐defined volumes. The average prostate volume difference of the 16 patients between the TCDR‐based and MRI‐defined volumes was 0.9 ± 7.3%.

**Figure 3:**
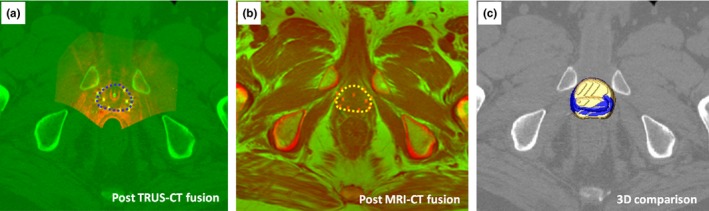
An example of TCDR‐defined and MRI‐defined prostate contours: (a) post TRUS‐CT fusion image where the TCDR‐based prostates contour is shown in blue, (b) the MRI‐defined prostate (yellow) in the post MRI‐CT fusion image, and (c) 3D comparison image of the TCDR‐based prostate volume (blue) and MR‐defined volume (yellow).

Figure 3 shows an example of the TCDR‐based and MRI‐defined prostate contour. Due to different patients’ positioning during TRUS, CT and MR scans, the prostate shape and orientation may vary on 3D, TRUS, CT and MR images. To compute the Dice overlap between the MRI‐defined prostate and our TCDR‐based segmented prostate, we registered the TRUS to CT images as well as the MR images to CT images.^22^ For all patients, the average Dice prostate volume overlap was 91.1 ± 2.3% between the TCDR‐based and the MRI‐defined prostate volumes. However, CT‐based prostate contours overestimated the prostate volume by 7.3 ± 18.7% as compared with MR‐defined prostate contours.

### 3.B Inter‐ and intra‐observer reliability of TCDR‐based prostate contours

**Figure 4:**
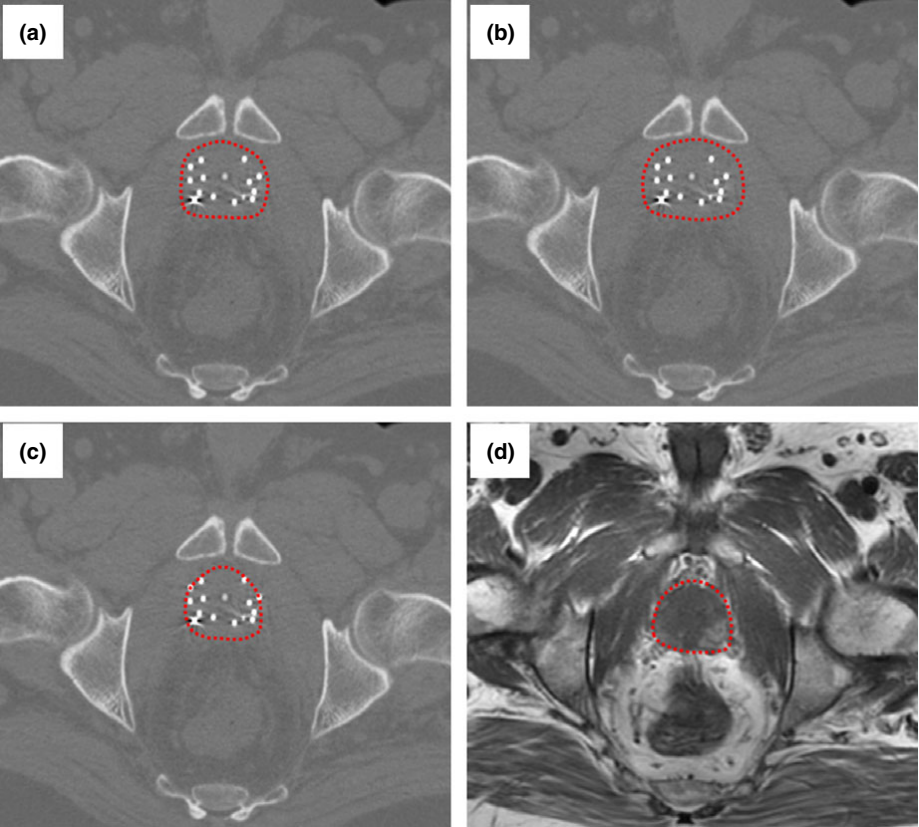
Inter‐observer CT‐based and TCDR‐based prostate contours: (a) physician 1 CT‐based, (b) physician 2 CT‐based, (c) TCDR‐based, and (d) MRI‐defined prostate contour (gold standard). The CT‐based contours (a) and (b) overestimate the prostate, and the TCDR‐based prostate contour (c) matches closely to the gold standard (d).

**Figure 5:**
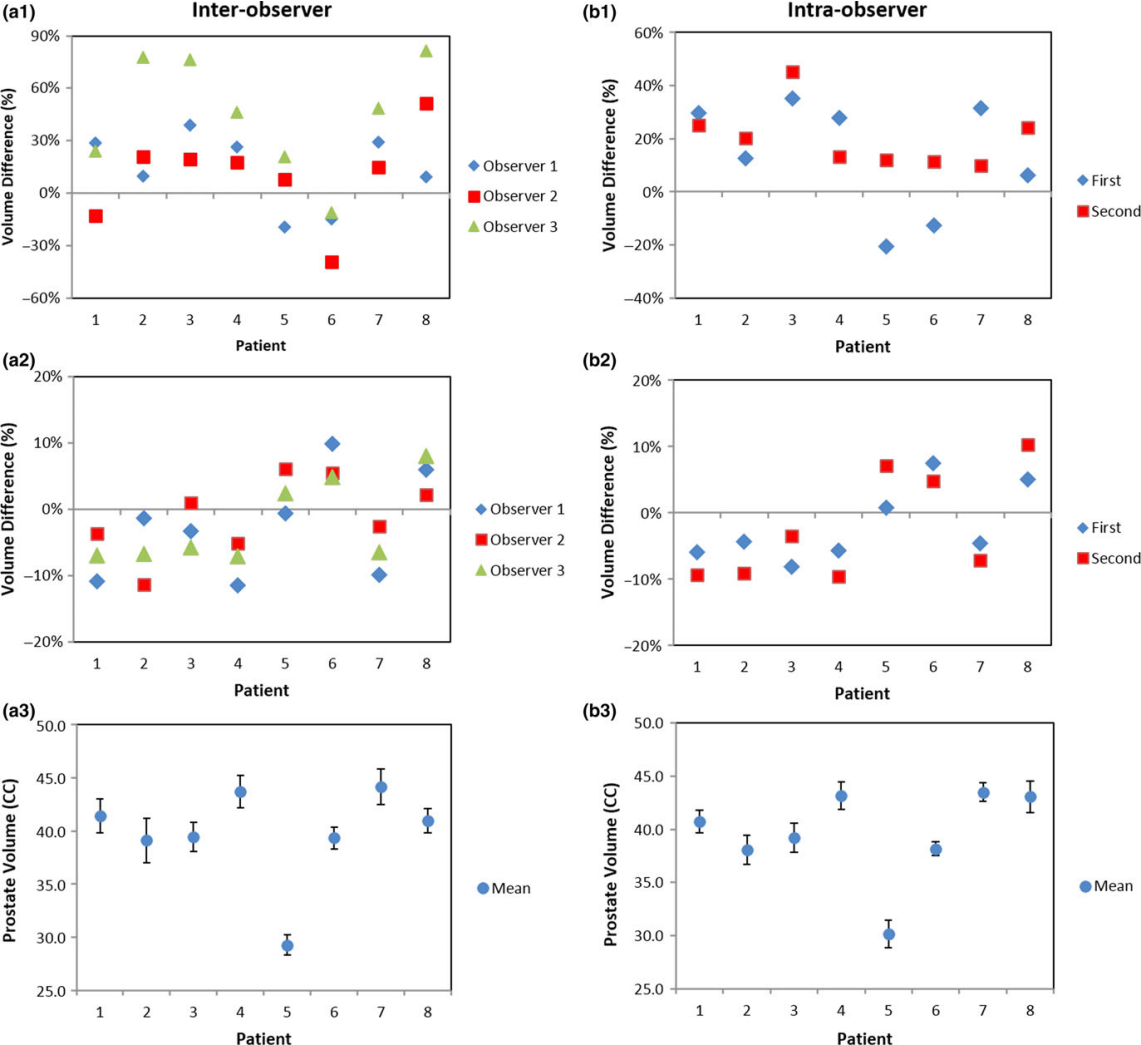
Inter‐ and intra‐observer reliability comparison of the prostate contours. (a1) The inter‐observer CT‐based prostate volume differences in three observers as compared with the gold standard MRI prostate contour; (a2) the TCDR‐based prostate volume difference; and (a3) the mean TCDR‐based prostate volume of 3 observers. (b1) The intra‐observer CT‐based prostate volume; (b2) the intra‐observer TCDR‐based prostate volume difference; and (b3) the mean intra‐observer TCDR‐based prostate volume difference.

Figure 4 shows an example of the TCDR‐based, conventional CT‐based (from two observers), and MRI‐defined prostate contours. Inter‐observer reliability of the prostate contours is demonstrated in Fig. 5(a). As shown in Fig. 5(a1), CT‐based prostate volumes tended to be larger than the MR‐defined prostate volumes for all three observers. The mean volume difference between the CT‐based prostate volumes and the MR‐defined prostate volumes for the 3 observers were 10.9 ± 28.7%, 13.7 ± 20.1%, and 44.7 ± 32.1%. There was a significant volume difference among CT‐based prostate volumes and the MR‐defined prostate volumes for two of the three observers (*P*‐values = 0.03, 0.02, and 0.01). With the TCDR‐based method, the mean volume difference between the TCDR‐based prostate volumes and the MR‐defined prostate volumes are 0.5 ± 7.2%, 1.8 ± 7.2%, and 3.5 ± 5.1% for the three observers, as shown in Fig. 5(a2). There are no significant prostate volume differences between the TCDR‐based segmentation volumes and the MR‐defined prostate volumes among the three observers (*P*‐values = 0.35, 0.30, and 0.18). The mean TCDR‐based prostate volume of the three observers is shown in Fig. 5(a3).

Intra‐observer reliability of the prostate contours is demonstrated in Fig. 5(b). Based on the TRUS prostate volumes segmented manually from the treating physician at the two different times, the mean volume difference between conventional CT‐based prostate volumes and the MR‐defined prostate volumes were 13.7 ± 20.1% and 20.3 ± 11.9% [Fig. 5(b1)]. With the TCDR‐based segmentation technology, the segmented the prostate volume difference between the TCDR‐based prostate volumes and the MR‐defined prostate volumes were 1.8 ± 7.2% and 2.2 ± 6.3% [Fig. 5(b2)]. There are no significant prostate volume differences between the two measurements by the same physician (*P*‐value = 0.45). The mean TCDR‐based prostate volume of the observer 1 is shown in Fig. 5(b3).

## 4 Discussions

**Figure 6:**
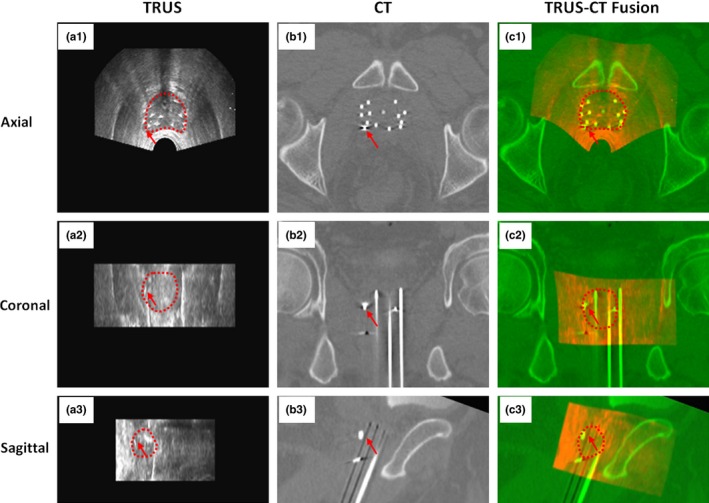
Example of TRUS‐CT registration. A gold marker (arrow) and catheters match well on the TRUS‐CT fusion image.

We developed a TCDR‐based HDR technology, which could significantly improve the accuracy of prostate delineation in prostate HDR brachytherapy. This TCDR‐based approach requires the acquisition of 3D intraoperative TRUS prostate images after the HDR catheter insertion which takes 1–3 minutes and can be easily integrated into the conventional HDR workflow. These TRUS images provide more accurate prostate contour than the CT images. This TCDR‐based HDR technology uses CT images for treatment planning because CT images provide clear visualization of catheter tips as well as accurate radiation dose calculation. Through TRUS‐CT fusion, the TRUS‐based prostate volume is transformed to the CT images for treatment planning. Specifically, the HDR catheters are reconstructed from the TRUS and post‐operative planning CT images, and subsequently used as landmarks for the TRUS‐CT image fusion. An example of TRUS‐CT deformable registration is shown in Fig. 6. Visually, the close match between the gold markers and the HDR catheters in the TRUS and CT demonstrated the accuracy of this TCDR‐based method. Note that the 10% difference in Dice coefficient means that the mean surface distance between the MRI‐defined and TCDR‐based prostate volume is around 0.5–1.0 mm for a typical prostate with 30–60 CC volume. The max surface distance between the MRI‐defined and TCDR‐based prostate volume is less than 2.0 mm for all patients. We anticipate that a margin of equal or larger than 2 mm would take care of this discrepancy.

The difficulties in defining the prostate contour using CT images are well‐known.^15^ Many CT prostate segmentation technologies have been investigated in recent years, such as the models‐based,^23^, ^24^, ^25^ classification‐based^26^, ^27^, ^28^ and registration‐based^29^, ^30^ methods. Most of these segmentation approaches are based on the appearance and texture of the prostate gland on CT images. However, in HDR brachytherapy the frequently used metal or plastic catheters introduce considerable artifacts to the CT images. These artifacts often smear the appearance and texture of the CT prostate images; therefore, these previous methods may not work well for the prostate HDR application. The prostate volume comparisons between the CT‐based and MRI‐based prostate contours of our study are in agreement with previous studies. In our study, the mean volume ratio between CT‐based prostate volumes and the MR‐defined prostate volumes ranged from 1.11 to 1.45, which is consistent with the results of 1.10 to 1.32 in the previous studies.^15^, ^16^, ^17^, ^31^

Many studies have shown that accurate prostate volumes can be obtained with both MRI and ultrasound.^15^, ^19^, ^32^, ^33^, ^34^ Traditionally, TRUS suffers from difficulties in distinguishing both the prostate apex and base, but the difficulty identifying margins may be alleviated using 3D TRUS technology.^15^ For instance, determination of superior and inferior borders can be assisted using reconstructed sagittal views. In addition, the smoothness of contours between adjacent axial slices could be improved, permitting observers to view reconstructed sagittal and coronal images. MR prostate imaging suffers from a lack of signal from cortical bone and image distortion near tissue‐air interface and fatty tissue, the soft‐tissue discrimination in MR images highly dependent on sequence. Our results show the mean ratios between TRUS‐base and MR‐defined prostate volume is about 1.04, which is consistent with the ranges (from 1.05 to 1.10) in the previously published studies.

In this study, we demonstrated that a 10 to 40% improvement in prostate volume accuracy could be achieved with the TCDR‐based approach as compared with the conventional CT‐based prostate HDR brachytherapy. The improvement of the proposed prostate delineation is resulted from the accurate TRUS‐based prostate contour as well as accurate registration between the TRUS and CT images. The registration between TRUS and CT images of the prostate is challenging, mainly because the anatomical structures in the ultrasound images are embedded in a noisy and low contrast environment with little distinctive information regarding the material density measured in the CT images. To deal with this challenge, we proposed a deformable registration method using HDR catheters as landmarks. In order to deliver a uniform dose to the prostate and spare the surrounding normal tissues such as the bladder and the rectum, the catheters were evenly placed to cover the entire prostate except the urethra. These HDR catheters provide exceptional landmarks to capture the non‐rigid prostate deformation between the TRUS and CT images. In addition, we chose a B‐splines transformation model, therefore, the translation of a point is only determined by the area immediately surrounding the control points to ensure locally controlled transformation. Because the deformations caused by a transrectal probe are spatially localized, this locally controlled transformation could be advantageous for registering TRUS images and result in smooth transformation fields.

There are several limitations to this study. First, it is the small number of patients. Nevertheless, we were able to demonstrate the significant improvement of the TCDR‐based prostate contour over the conventional CT‐based approach. Second, the diagnostic MR prostate images, which were used as the gold standard, were acquired prior to the HDR procedure. The prostate volumes might have changed between the MRI and HDR procedures for some patients who were receiving hormone therapy. Third, this is a retrospective study based on patients’ imaging data. Fourth, although our landmark‐based registration optimizes the global catheter match between CT and ultrasound, too many (more than 1/4−1/3 of the total number) catheter slips^35^, ^36^, ^37^ could affect the registration and TCDR‐based prostate contour accuracy. For future study, we plan to conduct a prospective clinical trial to further develop and refine the TCDR‐based prostate HDR brachytherapy.

## 5 Conclusion

Accurate delineation of the prostate is a key step to the success of HDR prostate brachytherapy. We have developed a new approach to improve prostate delineation utilizing intraoperative TRUS‐based prostate contour and TRUS‐CT deformable registration. In this study, we demonstrated its clinical feasibility and validated the accuracy with MRI‐defined prostate contours. This TCDR‐based HDR brachytherapy technology, which fits efficiently with the conventional HDR brachytherapy workflow, could improve prostate delineation, enable accurate dose planning and delivery, and potentially enhance prostate HDR treatment outcomes.

## Conflict of Interest

No conflict of interest.

## Supporting information


**Fig. S1.** Surface deference comparison.
