# Supplementary material for: Improved prostate delineation in prostate HDR brachytherapy with TRUS‐CT deformable registration technology: A pilot study with MRI validation
Source: J Appl Clin Med Phys. 2017 Jan 19;18(1):202–10. doi: 10.1002/acm2.12040 (PMC5689894; doi:10.1002/acm2.12040)
Supplement: Supplementary file 1 — Fig. S1. Surface deference comparison. [file ACM2-18-202-s001.docx]

**Ref.:  JACMP**

**[General Response]:** The authors wish to thank the reviewers and editor for the thorough review.

**Reviewer #3:**

How did you calculate that the mean surface difference is 0.5-1.0mm? I get a different number when I make a few assumptions. Assuming the dice coefficient is 0.9, and that one volume is exactly within the other volume, I solved the dice coefficient equation to find that 0.9 x Volume 1 = 1.1 x Volume 2, or Vol1 = 1.22xVol2. Then, assuming a spherical volume (V=4/3 pi r^3^), the ratio of the radii is 1.07. For a 30cc volume, this means that the difference in radii between the two volumes is 1.3mm; for 60cc the difference is 1.7mm. It still falls within the suggested 2mm margin, but the numbers here don’t match the ones given in the paper. Also, while the average is within the margin, what about the max discrepancy?

**[Response]:** The reviewer presented an estimation of the mean surface difference under a few assumptions. The following figure illustrates why there are discrepancies between the reviewer’s calculation and the result given in the paper. The surface difference between two segmented surfaces may be positive in some parts of surfaces and negative in other parts of surfaces. Thus, the mean surface difference value could be smaller than the value that the reviewer calculated. In this study we used commercial software (Amira) to calculate the mean surface difference. The max surface distance (discrepancy) is less than 2mm for all patients.

**Figure 1.** **Surface deference comparison.** The left figure is the spherical case that the reviewer used to perform the calculation of the absolute surface difference. The right is the real case that the reviewer used to perform the calculation of the mean surface difference.
